# Bringing non-human primate research into the post-genomic era: how monkeys are teaching us about elite controllers of HIV/AIDS

**DOI:** 10.1186/s13059-014-0507-y

**Published:** 2014-11-07

**Authors:** Eric J Vallender

**Affiliations:** Harvard Medical School, New England Primate Research Center, Southborough, MA 01772 USA

## Abstract

Whole-genome sequencing of Mauritian cynomolgus macaques reveals novel candidate loci for controlling simian immunodeficiency virus replication.

See related Research, http://genomebiology.com/2014/15/11/478

## Research highlight

Multiple factors influence the progression of an HIV-positive individual to acquired immune deficiency syndrome (AIDS); these include, their general health, the route of exposure and the specific HIV strain are a few examples of factors that can impact how long the virus remains latent in the body before the clinical manifestation of AIDS. Aside from these environmental factors, there also seem to be important host genetic contributions. Some individuals, termed ‘elite controllers’ or ‘long-term non-progressors’, can harbor the virus for an extended period of time without developing AIDS, even in the absence of treatment. Understanding the underlying genetic changes that define these individuals could suggest new treatment strategies or improve the development of vaccines.

Animal models minimize the contribution of environmental factors and can thus exaggerate genetic effects. This makes them ideal for identifying genetic causes of complex phenotypes. In this issue of *Genome Biology*, Ericsen and colleagues use whole-genome sequencing of Mauritian cynomolgus macaques (*Macaca fascicularis*) to identify candidate loci affecting the control of simian immunodeficiency virus (SIV) [1]. Using animals from previous work, whole-genome sequencing enabled the identification of seven candidate control regions that merit further study. Perhaps more importantly, however, the work demonstrates the utility of whole-genome sequencing in non-human primates in several important respects: for improving our understanding of the genetic basis of disease, for refining animal models, for increasing translational meaning and for reducing unnecessary studies.

## An animal model of AIDS

In 1982, veterinarians at the New England Primate Research Center recognized an increase in the number of deaths associated with immunosuppression in their macaque colonies. As they reviewed the case-history for these animals, it became apparent that there were strong similarities between the disease they were seeing and the burgeoning AIDS epidemic [2]. At the time, the etiologic agent of the disease, in humans and in macaques, was unknown. This would change quickly; in the next two years, researchers successfully isolated a T-cell-tropic retrovirus from macaques that had died from this AIDS-like disease [3]. Shortly thereafter, they were able to induce the disease state in macaques through inoculation with the virus, conclusively identifying the agent of the disease and developing the first animal model of human AIDS [4]. Since those early years, macaques have become the best model of HIV/AIDS and have greatly furthered our understanding of the disease [5].

SIV is part of a broader group of lentiviruses that includes feline, bovine, ovine/caprine, and equine relatives [6]. Over 40 species of African primates are endemically infected with various strains of SIV. The most notable examples are chimpanzees and sooty mangabeys, in which HIV-1 and HIV-2, respectively, originated. In their natural hosts, the SIV viruses are generally not pathogenic (SIV_cpz_ is a notable counterexample); this is due to coevolution of the virus and host immune systems [7]. However, when these viruses cross species boundaries into non-native hosts, the pathogenic effects emerge. This is what has happened with the introductions into humans and is also what we observe in the Asiatic macaques. The SIV that affects these macaques, like HIV-2, seems to have originated from an endogenous sooty mangabey virus.

The Asiatic macaques, including both the commonly used rhesus macaque (*Macaca mulatta*) as well as the cynomolgus macaque *M. fascicularis*, are useful models for human HIV infection and progression to AIDS because they too are not naturally occurring hosts, are susceptible to infection by closely related viruses and show similar symptomology and disease progression to that in humans. These factors mean that they are useful not only for understanding host-virus interactions but also for studying post-infection treatment and vaccine-development options. Together, this places an incredible importance on non-human primate models for HIV/AIDS research that cannot be recapitulated in other model organisms (such as rodents), in cell-culture systems or in humans.

## An audience with the Red Queen

Over the past two decades, a number of host genetic factors have been identified that affect susceptibility to infection with HIV and disease progression. This includes both genetic factors in natural hosts, which ultimately result in the non-pathogenicity of their resident viruses, as well as those in humans and macaques that impact the course of disease. In early studies, alleles for human leukocyte antigen (HLA) were identified that influence the course of HIV infection [8]. The pervasive importance of HLA alleles in infectious disease has long been well established, and this signal is regularly and consistently observed in association with diseases with an infectious or inflammatory component. Since then, a number of additional host genes have been identified, with differing levels of confidence (reviewed in [9]).

Identification of genetic factors has largely come either from human genome-wide association studies (GWAS) or from candidate-gene studies in non-human primates. For HIV/AIDS, the same issues that regularly plague human GWAS are found; few loci reach genome-wide significance, and those that do tend to recapitulate previously known effects (notably HLA) without many unambiguous novel findings. Candidate-gene studies in non-human primates have been more useful in identifying novel targets. These have largely made use of our existing knowledge of the mechanisms by which SIV enters the cell and through extrapolations from cell-culture experiments. Until now, these two approaches had not intersected.

In this month’s paper, Ericsen and colleagues [1] offer the first application of whole-genome sequencing to interrogating SIV progression in macaques. Their genome-wide analysis offers seven novel candidate loci for host control of SIV replication, identifying unique and previously uninterrogated regions for further study. Taken alone, this represents seven potential new targets for therapeutic development and seven potential new footholds for furthering our understanding of disease. With the devastation wrought by HIV/AIDS, this represents a potentially meaningful advance by itself. It is equally noteworthy, however, that this represents a new step forward more broadly.

Two scientific criticisms of non-human primate research are consistently voiced, especially by those used to dealing with rodent studies: first, that monkeys are outbred and genetically heterogeneous (which introduces genetic variability, and makes mapping difficult); and second, that, for a variety of reasons, sample sizes are often small. This study takes advantage of the genetic variation inherent in cynomolgus macaques to identify novel loci in a forward-genetic approach. The lack of an existing comprehensive map of genetic variation makes whole-genome sequencing necessary but, in turn, ensures a minimum of *a priori* bias. Cleverly, the authors first segregate animals by major histocompatibility (MHC) type, both offering an internal positive control for their methodology and eliminating a previously known control locus whose signal has the potential to drown out any new findings. As a result of this care, as well as the substantial environmental control afforded by animal studies, genetic-effect sizes are maximized and the small study size is not prohibitive. Taken as a whole, this study demonstrates the power of non-human primate studies and shows how common criticisms can be overcome, even in whole-genome studies.

## A wheel still in spin

The work by Ericsen and colleagues [1] represents the first actual implementation of a future that many in the non-human primate genetics community have long envisioned [9,10]. It brings non-human primate research into the post-genomic era, finally fully realizing the advantages of the model. It also builds upon decades of previous non-human primate research on SIV. Over the years, the macaque SIV model has seen extensive use in studies of the basic biology of the disease as well as in vaccine and treatment development. For many of these studies, detailed records that describe the differing responses of individual animals are available, as well as blood, tissue samples or isolated genomic DNA. Now that whole-genome sequencing is finally a reality, these historic studies can be subjected to modern and rigorous genetic interrogation.

The new work [1] offers a guidepost to how this might be done. It carefully selects animals *a priori* on known genetic factors and on previously determined responses to carefully controlled experiments. By measuring the density of heterozygous variation between groups that differed in their ability to control viral replication after 52 weeks, it was possible to identify regions where the two groups differed and where, potentially, there might exist novel host control factors. In the future, this approach can be expanded and adapted to achieve greater power.

While this study separates elite controllers from standard progressors in the search for host determinants of SIV replication, it could just as easily be applied to other disparate classes of animals: those with behavioral abnormalities, those that respond differently to pharmaceuticals or drugs of abuse, or those that show more pronounced cognitive decline with aging. The entire realm of non-human primate models for which there exist genetic components is potentially amenable to this approach. Specifically, in the context of understanding host control of SIV, sequencing of additional animals will further narrow and refine the list of candidate genetic loci and, as additional functional genetic regions are identified, further *a priori* segregated groups can be studied. Finally, of course, as more animals that are suitable for genetic follow-up studies are identified, other methodologies for dealing with the whole-genome sequencing data will become available.

This moment has long been on the horizon, a realization of the promise of the post-genomic era for understanding the genetic causes of complex disease. In human studies, this vision has generally proven illusory as a result of environmental variation and uncontrolled, or uncontrollable, variables, save for rare exceptions. Now, however, Ericsen and colleagues have produced a practical, workable, approach utilizing non-human primates [1]. The road forward is clear, and it is now only up to the research community to take advantage.

## Abbreviations

AIDS: Acquired immune deficiency syndrome
GWAS: Genome-wide association study
HIV: Human immunodeficiency virus
HLA: Human leukocyte antigen
MHC: Major histocompatibility complex
SIV: Simian immunodeficiency virus
